# Gamified Cognitive Control Training for Remitted Depressed Individuals: User Requirements Analysis

**DOI:** 10.2196/games.8609

**Published:** 2018-04-05

**Authors:** Jasmien Vervaeke, Jan Van Looy, Kristof Hoorelbeke, Chris Baeken, Ernst HW Koster

**Affiliations:** ^1^ Psychopathology and Affective Neuroscience Lab Department of Experimental Clinical and Health Psychology Ghent University Ghent Belgium; ^2^ imec - Research Group for Media, Innovation and Communication Technologies - Ghent University Ghent University Ghent Belgium; ^3^ Department of Psychiatry and Medical Psychology Ghent University Hospital Ghent Belgium; ^4^ Department of Psychiatry University Hospital Brussels Brussels Belgium; ^5^ Ghent Experimental Psychiatry Lab Ghent University Ghent Belgium

**Keywords:** depression, cognitive dysfunction, cognitive remediation, relapse prevention, qualitative research, secondary prevention

## Abstract

**Background:**

The high incidence and relapse rates of major depressive disorder demand novel treatment options. Standard treatments (psychotherapy, medication) usually do not target cognitive control impairments, although these seem to play a crucial role in achieving stable remission. The urgent need for treatment combined with poor availability of adequate psychological interventions has instigated a shift toward internet interventions. Numerous computerized programs have been developed that can be presented online and offline. However, their uptake and adherence are oftentimes low.

**Objective:**

The aim of this study was to perform a user requirements analysis for an internet-based training targeting cognitive control. This training focuses on ameliorating cognitive control impairments, as these are still present during remission and can be a risk factor for relapse. To facilitate uptake of and adherence to this intervention, a qualitative user requirements analysis was conducted to map mandatory and desirable requirements.

**Methods:**

We conducted a user requirements analysis through a focus group with 5 remitted depressed individuals and individual interviews with 6 mental health care professionals. All qualitative data were transcribed and examined using a thematic analytic approach.

**Results:**

Results showed mandatory requirements for the remitted sample in terms of training configuration, technological and personal factors, and desirable requirements regarding knowledge and enjoyment. Furthermore, knowledge and therapeutic benefits were key requirements for therapists.

**Conclusions:**

The identified requirements provide useful information to be integrated in interventions targeting cognitive control in depression.

## Introduction

### Background

In 2010, 7.4% of the total worldwide disease burden was attributed to mental and substance use disorders, of which depressive disorders accounted for the most disability-adjusted life years [1]. As such, major depression is recognized as a global leading cause of disability [2]. One of the key challenges within this context is that existing treatments frequently fail to achieve stable remission (for an overview, see [3]). Furthermore, recurrence and relapse rates are estimated to be 54% in the second year after receiving therapy [4], which increases with the number of previous depressive episodes [5,6]. As a result, preventing recurrent major depression is of paramount importance.

In this context, research suggests that impairments in cognitive control (eg, [7-9]), which refer to difficulties in executive processes such as shifting, inhibition, and updating of information in working memory [10], place one at an increased risk for recurrence of depressive episodes (eg, [11,12]). For instance, research indicates that impaired cognitive control hampers effective emotion regulation and task engagement [13,14]. Furthermore, remitted depressed (RMD) individuals are still suffering from these impairments (eg, [15,16]), where cognitive control deficits are related to the number of past episodes [17]. Interestingly, recent evidence suggests that traditional therapies leave these cognitive risk factors for depression unaffected, even when effectively reducing symptoms of depression [18]. These observations have led to the development of cognitive control training (CCT) for depression.

### Improving Cognitive Control

The idea behind CCT is that performing it activates the prefrontal cortex repetitively, which improves attention and cognitive control (for reviews, see [19,20]). In the past decade, CCT has been used in several studies regarding vulnerability for depression, and results overall seem promising (eg, [21,22]). CCT has been shown to have additional effects to treatment as usual, in terms of rumination and depressive symptomatology [19]. In a 1-year follow-up of this study, CCT also demonstrated long-term potential [23]. Other studies showed that CCT in depression is promising in combination with other treatments, for instance, with transcranial direct current stimulation [24]. Interestingly, there are some studies that have found that stand-alone CCT also has effects on rumination [21,22,25,26] and depressive symptomatology [27]. A recent meta-analysis of computerized cognitive training for depression showed that it has a small to moderate but nevertheless consistent effect on depressive symptomatology and everyday functioning [28]. Moreover, a recent randomized controlled trial (RCT) showed that CCT, compared with an active control condition, is effective in training cognitive performance and yielded significant differences in terms of rumination, depressive symptomatology, and functioning in an RMD sample [29]. Importantly, effects remained stable over time (at 3-month follow-up).

Provided the encouraging findings of well-controlled studies using CCT and the potential to reach a wider population of at-risk individuals through Web-based administration, the CCT program needs to be suitable for Web-based administration. The CCT studies presented above used the Paced Auditory Serial Addition Task (PASAT; [30]). In this task, participants hear a stream of digits, with a certain interstimulus interval (ISI). Participants have to calculate the sum of the last 2 digits and click on the corresponding number. In an adaptive variant of the PASAT (aPASAT), the ISI decreases after a certain number of consecutive correct responses and increases after a certain number of consecutive incorrect responses. However, given that the task is not enjoyable, its user experience is problematic (eg, [31]). The task is rather monotonous and demanding, and it can be frustrating at the same time [23,26,32]. The second problem with CCT is that participants have to complete several sessions to benefit from the training (ie, they have to visit the research laboratory, hospital, or Web-based platform multiple times), which can easily lead to dropout in both research contexts and treatment settings. Indeed, dropout numbers in CCT studies can be substantial. In a selection of 10 influential CCT studies [19,24,26,27,29,33-37] in the context of depression vulnerability, mean dropout was at least 21%, ranging from 4% to 50%. If we take sample size into account, total dropout was at least 234 participants of 1697 over the 10 studies, hence 13.79%. Fortunately, enhancing treatment credibility shows potential to counter dropout [38] and improve treatment effects [39]. That is, user engagement has been identified as a crucial variable for mobile health apps [40] and CCT procedures [23], where increasing user engagement may thus be of paramount importance to increase effects of CCT interventions.

### Increasing User Engagement

One of the ways to achieve this is by means of *gamification* [41]. *Gamification* refers to the addition of game elements to nongame contexts, such as points, levels, or a narrative. By increasing engagement, users tend to do better at tasks and rate them more fun at the same time [42]. However, gamification can be implemented in various ways. There are a lot of different game features, and each can have a different effect on a different target population. It has been used in interventions for common mental disorders such as depression, but it is unclear which gamification elements act most effectively [43]. Moreover, little is known about game and gamification preferences of RMD individuals. Provided that patient preferences can influence therapy outcomes (eg, [44]), gamification elements need to be suitable and motivating for RMD individuals. Recently, a call was launched to increase adherence and decrease dropout rates in internet interventions through, among other things, increasing user engagement and adding gamification elements [45]. In addition, CCT should fit within participants’ daily routine. To achieve these goals, a *user requirements analysis* was conducted.

A *user requirements analysis'* goal is to understand the needs and requirements of the intended users [46]. Conducting such an analysis can yield several benefits such as improved user satisfaction, increased productivity, and reduced costs for training and support [47]. According to the study by Robertson and Robertson [48], there are different kinds of stakeholders to consider in a user requirements analysis. In our case, the intended beneficiaries of training (operational working area) are RMD individuals. Another group of stakeholders are those who might benefit from the training without being directly in contact with it (containing business): mental health care professionals (MHPs), who can play a key role in prescribing and monitoring CCT. This analysis will therefore regard the requirements of these 2 stakeholder groups.

### Aim of the Study

This study is a first step in developing a new intervention targeted at RMD individuals as a relapse prevention program, through CCT. To increase uptake and adherence, a user requirements analysis was considered crucial.

The aim of this study was to explore and map the needs and preferences of an RMD sample and an MHP sample. More specifically, we have 4 research questions. Questions 1 and 2 were queries for the RMD population. Questions 3 and 4 were key questions examined in the group of MHPs.

1. What are the mandatory basic requirements to start CCT?
2. How can user engagement and adherence to CCT be optimized?
3. What are mandatory basic requirements to implement CCT in treatment?
4. How can implementation of CCT in treatment be facilitated?

## Methods

### General

To receive feedback regarding the aPASAT and the broader platform, a user requirements analysis was conducted. To this end, we organized a focus group for RMD individuals, seeing that this is a cost-effective method for analyzing user requirements [49]. To best suit the busy schedules of the clinicians, we conducted individual interviews with them. All focus groups and interviews were conducted in Dutch and were semistructured, based on a topic list (see section Materials). Citations in this paper have been translated as literally as possible. Names have been changed to guarantee anonymity.

### Participants

#### Remitted Depressed Individuals

Five participants were included in this focus group. The sample consisted of 3 men and 2 women, and their ages ranged from 32 to 62 years (mean 48.4, SD 9.9). All had suffered from at least 2 depressive episodes in the past and were in remission at the time of the focus group. Three participants (1 woman) also participated in an RCT study regarding CCT in the previous year [29] and were selected as part of a follow-up from a larger sample pool. In addition, an organization for patients with depression was contacted through email and yielded the other two participants with no experience regarding CCT. All participants indicated to have a moderate to high affinity for technology as measured by the short version of the Affinity for Technology Scale (ATS) [50,51]. They were rewarded with 25 euros.

#### Mental Health Care Professionals

Six MHPs agreed to be interviewed. All received a psychology degree from a Belgian university. Mean age was 36.7 years (SD 13.1) and ranged between 26 and 65 years. Of the 6 experts, 2 were females. Three participants worked as psychologists, one had recently retired but used to work as a psychologist, one was a researcher who also works as a psychologist with patients, and one was a researcher specialized in electronic and mobile apps for mental health care. The last 2 held a doctoral degree. All participants indicated to have a moderate to high affinity for technology as measured by the short version of the ATS. Participants were recruited via email. We focused on contacting therapists using a cognitive (behavioral) approach, as well as recruiting professionals from different working environments (eg, hospital, private practice, and mental health centers). Participants received an incentive of 15 euros.

### Materials

Both the focus group and the interviews were recorded on 2 audio recorders, placed at 2 different locations on the table around which everyone was seated. For the aPASAT demo, a standard Dell laptop (Dell Technologies Inc, Round Rock, Texas, USA) running Windows 7 (Microsoft Corporation, Redmond, Washington, USA) was used. The focus group and the interviews had a distinct topic list because these were conducted with different target groups. These lists were created in advance by the first author (JV), based on the research questions, and sent out to 2 coauthors (JVL and EHWK) for adaptation and approval. These lists were not changed during the focus group or interviews, but the order was flexibly adapted. However, all topics were covered.

### Procedure

#### Focus Group

**Figure 1:**
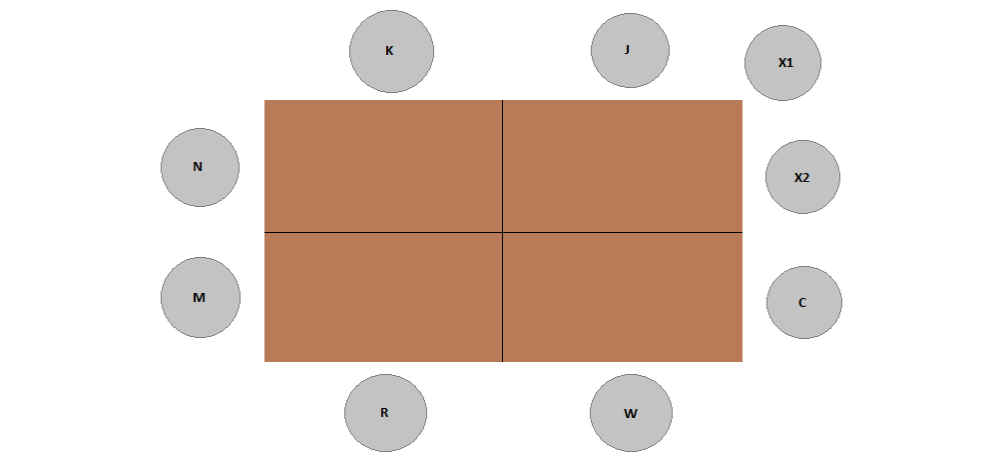
Seating arrangement of the focus group. Karl (K), Jeff (J), Chris (C), Will (W), and Rachel (R) were participants. M, N, X1, and X2 were moderator, note taker, researcher from the randomized controlled trial (RCT) study, and an employee of a game developer company, respectively.

Participants completed informed consent, a demographic questionnaire, and the ATS. The seating arrangement is illustrated in Figure 1. The focus group included a demo of an aPASAT session that was used in previous studies (eg, [29]). At the end of the focus group, participants received their incentive. The whole procedure lasted about 150 minutes and took place at the Faculty of Psychology and Educational Sciences of Ghent University. The group was moderated by an investigator with extensive experience with focus groups (JVL).

#### Mental Health Care Professional Interviews

Participants completed informed consent, a demographic questionnaire, and the ATS. The interview started with broad, easy-to-answer questions as an icebreaker. After this, the aPASAT demo was presented, and the interview core questions were asked, each followed by probing questions or requests to elaborate. The interviews ended with the incentive. Duration of the interviews ranged from 45 to 90 minutes. Given the busy schedule of interested therapists, the interviews took place at a location of their choice. This resulted in 3 interviews in the work setting, 2 at the faculty, and 1 at home. The interviewer did not have previous interview experience. However, literature regarding interviews was consulted, and 2 test interviews were conducted with researchers, experienced with interviews.

### Data Analysis

All conversations were recorded, transcribed, and analyzed using NVivo (QSR International, Melbourne, Australia), a software package for qualitative data analysis, using an inductive thematic analytic approach (as described in [52]). The coding took place in three broad stages, led by JV, in close collaboration with JVL and EHWK in the third stage. First, transcriptions were given a code as close as possible to the transcription. Here, text segments could have none, one, or multiple codes, and there was overlap between codes. Second, the codes were compared and analyzed. This was done code by code. In this stage, similar codes were merged, and codes with multiple meanings were separated so that each code had only one meaning and a clear coverage that fitted all corresponding text segments. Third, codes were grouped into categories, and different structures were imposed and discussed. Results show the final chosen structure.

## Results

### General Information

The results are organized in relation to each of the 4 research questions proposed earlier. Furthermore, each section includes a table that reflects the main findings. Finally, some pitfalls and opportunities are added at the end.

### Mandatory Requirements for Remitted Depressed Sample

Table 1 reflects the mandatory requirements for an RMD sample, which is largely self-explanatory. Patients indicated that a calm and private environment is necessary to focus on the training, but they do not want to feel isolated. Jeff (male, 52 years old, RMD individual) explained how demanding the PASAT can be:

When I did the sessions at home, the only time I could complete them was when my children were sleeping and when I asked my girlfriend not to talk for 20 minutes.

### Desirable Requirements for Remitted Depressed Sample

Features that might help RMD individuals to adhere to CCT are written down in Table 2. Psychoeducation is considered crucial, given that face validity is quite low, and it should emphasize that (1) the main aim of the training is to reduce depressive symptomatology and chances of relapse and (2) the importance of completing the intervention. But when doing so, it should be specific and concrete, as expressed by Rachel (female, 45 years old, RMD individual):

I believe you need to be clear, in order to motivate people to keep doing it, to be really clear what it will yield.

Participants further agreed that response-related feedback during training is important. This should ideally be clear, instant, and easy to process (in other words, it should not interfere with the ongoing task). Patients reported that in many contexts, feedback is important to improve task performance, whereby improvement itself can be a motivating element in the words of Karl (male, 32 years old, RMD individual):

The first session, I was searching. The second one, I was cursing. The third one I was cursing some more, but from the fourth session on, I found it agreeable. Why? Because I noticed I was getting better. So, it is fun, to notice your own progression.

**Table 1:** Overview of the mandatory requirements for the remitted depressed (RMD) individuals. CCT: cognitive control training. PC: personal computer. FAQ: frequently asked questions.

Category and subcategory		Requirement	Implementation
**Technology**			
	Functionality	Software works, no bugs, no crashes	Develop bug-free CCT
	Usability	Software should be user-friendly	Easy to use; simple text; visually appealing; available on PC and tablet
**Person**			
	Skills	Some internet and technical knowledge	State clear expectations; devise good manual; write clear FAQ section
	Access	Internet connection, device in possession	CCT is compatible with most browsers; both on PC and tablet
**Configuration**			
	Time	Not too long and flexible planning of sessions	15 minutes per session; calendar tool to plan next session
	Location	Flexible	CCT on an internet website
	Setting	Calm and private, but not isolated	Including tablets enables users to complete sessions at preferred location
	Pricing	For free	Available for free

**Table 2:** Overview of the desirable requirements for the remitted depressed (RMD) individuals. CCT: cognitive control training. FAQ: frequently asked questions.

Category and subcategory		Requirement	Implementation
**Intrinsic motivation**			
	Psychoeducation	Knowledge about CCT mechanism and expected outcomes, preferably in an interactive manner	Provide psychoeducation that is clear, simple, and interactive
	Practical or technical assistance	Practical and technical guidelines regarding CCT and protocol in case of problems	Devise FAQ section; communicate contact options
	Gamification	Training should be enjoyable, engaging, and challenging, by including feedback and reinforcing messages	Include performance-dependent feedback; include performance-independent stimulating messages; individualize CCT
**Extrinsic motivation**			
	Incentivization	Monetary reward after completing all sessions or monetary penalty when dropout	Will not be implemented; available for free
	Motivation by therapist	Rationale, follow-up, and encouragement by the therapist when asked for or needed	Therapist will play a role in dissemination and administration, but options not clear at this point

Another gamification element that patients agreed on was simulated social reinforcement, which can be seen as a kind of reward. Social reinforcement is a form of positive reinforcement and an umbrella term for getting approval from others, by means of attention, praise, and encouragement, among others, as shown in the example of Chris (female, 50 years old, RMD individual):

[When playing Candy Crush Saga]…there is a little figure jumping up and down and telling me “Well done!,” which I enjoy hearing once in a while. Or you can hear “Sweet!,” which I also enjoy hearing.

The therapist could also play a role in adhering to the training and, more interestingly, this was also offered in the interviews with MHPs; thus, it certainly is considered possible to fulfill this role. Moreover, some MHPs even regarded motivating patients their responsibility, seeing how a therapist’s opinion and presentation of a treatment can impact a patient’s reception and motivation.

#### Mandatory Requirements for Mental Health Care Professionals

Table 3 lists the mandatory requirements for a sample of MHPs. As in the previous paragraph with mandatory requirements, this is rather straight-forward.

#### Desirable Requirements for Mental Health Care Professionals

Features that facilitate implementation of CCT are listed in Table 4. Educating therapists with regard to the training, as well as explaining how and why it works, is considered important for dissemination, especially given the low face validity of this training, as Rudy (male, 65 years old, psychologist) said:

When I imagine my colleagues, I think there are certainly some, because they regard it as hocus-pocus, that are not willing to offer it [the training].

**Table 3:** Overview of the mandatory requirements for the mental health care professionals (MHPs).

Category and subcategory		Requirement	Implementation
Permission		Need permission from supervisor or MHP	Get approval by showing scientific evidence; giving psychoeducation
**Configuration**			
	Time	Offering training should not be time-consuming	Devise training to be minimally time-consuming for MHP
	Usage	Be informed about practical information of the training	Provide clear guidelines; practical or technical assistance (eg, contact person)
	Format	Available and shareable training	Freely available website (not CD-ROM or other physical carriers)

**Table 4:** Overview of the desirable requirements for mental health care professionals. CCT: cognitive control training.

Category and subcategory		Requirement	Implementation
**Intrinsic motivation**			
	Psychoeducation	Knowledge about CCT mechanism and expected outcomes	Provide clear psychoeducation; tailor it to the working environment
	Therapeutic benefits	CCT is beneficial in preventing or postponing a relapse; without major side effects	Test effectiveness of the CCT extensively; check for side effects
**Extrinsic motivation**			
	Resource access	Having access to scientific papers or clinical tools through the university	Unfortunately not possible to implement
	Training feedback	Information about execution, performance, and outcome of the patients they proposed CCT to	Unclear at this point; if possible, grant therapist restricted access to database

It might be encouraging to receive training feedback as well as outcomes with regard to depressive complaints, which would allow seeing whether training is effective for specific patients. Therapists indicated that such information could increase motivation to implement training, as Hailey (female, 26 years old, and psychologist) stated:

If we, as caregivers, are able to check whether or not they completed a session, or that you [the researchers] would be able to send us a message, for instance “We noticed that this person is not really strict with completing the sessions,” we might be able to call that person and really motivate [him/her], like, “Really try to do it anyway” and explain again why exactly it is a good idea.

In the example above, it is also clear that MHPs are willing to take on a motivational role, as discussed in the previous section.

### Pitfalls

RMD individuals and MHPs raised a number of additional concerns and comments that could be taken into account to prevent problems with the training. We will briefly present seven such concerns. First, the PASAT itself is based on math operations, which can be frustrating for some people, especially when time pressure is added. Second, privacy and data protection is considered important.

Third, technological skills will vary where it is important to strike a balance for being easy to operate for novice as well as skilled users. Fourth, the amount of additional work in addition to the training (such as questionnaires) needs to be limited. Fifth, having the training publically available means that nobody gets excluded this way, but this comes with the risk of offering the training to relapsed individuals that will not be helped by the training on its own, resulting in a fail experience, further decreasing this person’s mood. Sixth, the effect of the training is the strengthening of cognitive control, which is hard to measure and notice and comes only after multiple training sessions, but users might be focused on their performance which is easily measured and visible. Finally, gamification might lead to extra frustration. Having too many gamification elements within the training, so that it distracts from the task, should be avoided.

### Facilitating Factors

Facilitating factors are already existing opportunities which might support the uptake of and adherence to this training. First, depression and relapse rates are high, ensuring our training has a large potential user base. Second, in clinical practice, RMD individuals are requesting preventative programs. There are some relapse prevention programs (eg, mindfulness-based interventions), but the need for targeted interventions for cognitive vulnerability remains. However, RMD individuals sometimes take initiative themselves. Some of them will play brain training or attention games on their own. Here, it is safe to say that the step to our training would not be huge, and these individuals are motivated to prevent a relapse. Finally, some therapists still have follow-up sessions with RMD individuals, so offering the training through the therapist might be a useful strategy.

## Discussion

### Principal Findings

The aim of this study was to conduct a user requirements analysis with RMD individuals and MHPs to take into account their preferences in the next steps of development of Web-based CCT, aimed at relapse prevention in depression. For this purpose, we conducted a focus group with RMD individuals and interviewed MHPs. We performed qualitative analyses on the input provided by these participants. Finally, we identified some hindering and facilitating factors that can influence uptake of and adherence to the training.

For the RMD group, there were several mandatory requirements: the training should be functional and user-friendly and match this group’s technological skill and access. Performing the training sessions should also fit within their daily routine. Furthermore, this group desired the training to be engaging. Finally, they can be motivated by knowledge of how training works (ie, psychoeducation), training progress, gamification elements in the training, external factors, and their therapist.

The MHP group postulated some key requirements as well. Offering the training should be approved by their supervisor or director when they are working in a mental health care center. When they are self-employed or need supervisor approval, scientific arguments and research showing the effectiveness of the training and explaining how the training works are needed. Psychoeducation and feedback about patients’ progress can increase the chance that therapists will offer this training.

This study has 2 main strengths. First, as the endpoint of this project is creating an intervention for RMD individuals, we actually involved these end users, as well as MHPs who are in frequent contact with this population. This is in accordance with a recent call by an international collaboration, called “COMETS” (Collaboration On Maximizing the impact of E-Therapy and Serious gaming). COMETS was created to increase the uptake and adherence to internet interventions for mental health [45]. They plead for a paradigm shift that is built on 4 pillars: (1) increased focus on users, (2) increased focus on engagement, through gamification or other techniques, (3) more intersectoral collaboration, and (4) rapid testing and implementation. In this study, it should be apparent that we tried to comply with the first 2 pillars. Furthermore, we will also attempt to meet the other 2 by (1) collaborating with software developers who have more experience with good usability, design, and dissemination; (2) conducting research that tests the training’s effectiveness as well as the effect of gamification; and (3) thinking about implementation of the training in the early stages of development. By taking into account all these factors, including the results of this study, we hope to decrease dropout rates of CCT and deliver a widely available Web-based intervention.

A second strength is that our findings are in agreement with literature regarding internet-based cognitive behavior therapy, which adds to the reliability of the findings of this study. For instance, previous research has shown beneficial effects of an intuitive and interactive product [53], of reminders and motivational prompts [54], and that presented text should match the individual’s reading and computer skill [55]. A study by Rozental et al [56] showed a decrease in motivation for a Web-based intervention when the amount of mandatory reading was too high, as this was too time-consuming.

### Limitations

Nevertheless, this study also has some limitations. First, both target populations were represented by a small sample. Findings can therefore not be easily generalized, but it does give us some specific insights. During the focus group with only 5 RMD individuals, opinions already diverged greatly. Although relatively few people were included, enrolling too many people in a focus group might work counterproductive [49]. Furthermore, this study counts as a first step. In subsequent research, user experience and engagement will be monitored in larger samples. The generalizability of this study might be rather low; however, the main aim here was to go into depth, which was not hindered by our final sample size. Given these limited samples, we do not assume saturation has been reached. Nevertheless, we believe that the most crucial aspects have been identified.

As a second limitation, because some of the questioned variables are a matter of personal preference, we did not go into detail about those in this study. For instance, Bartle [57] identified 4 player types, that each has their own motivation of why gamers play. Different player types are appealed to different game mechanics. Making the distinction would certainly have offered interesting viewpoints. However, as we are aiming to reach the whole RMD population, instead of specific subgroups, we chose not to include this or other distinctions.

### Future Steps

Future steps of this project will be to develop the software of the training and platform, based on the input from the RMD individuals and the MHPs, after which it will be tested in several experiments to ensure that the training is efficacious and can be disseminated. The present requirements analysis is a crucial step in ensuring that the target population will be motivated and engaged to perform the cognitive control program.

## Abbreviations

aPASAT: adaptive PASAT
ATS: affinity for technology scale
CCT: cognitive control training
COMETS: Collaboration On Maximizing the impact of E-Therapy and Serious gaming
ISI: interstimulus interval
MHPs: mental health care professionals
PASAT: paced auditory serial addition task
RMD: remitted depressed
